# Supplementary material for: Splice-Junction-Based Mapping of Alternative Isoforms in the Human Proteome
Source: Cell Rep. Author manuscript; Available in PMC 2020 Jan 15. (PMC6961840; doi:10.1016/j.celrep.2019.11.026)
Supplement: 3 [file NIHMS1546469-supplement-3.zip › DF2/PXD0010154/Lung-172-P15502-YGVAARPGFGL.pdf]

A

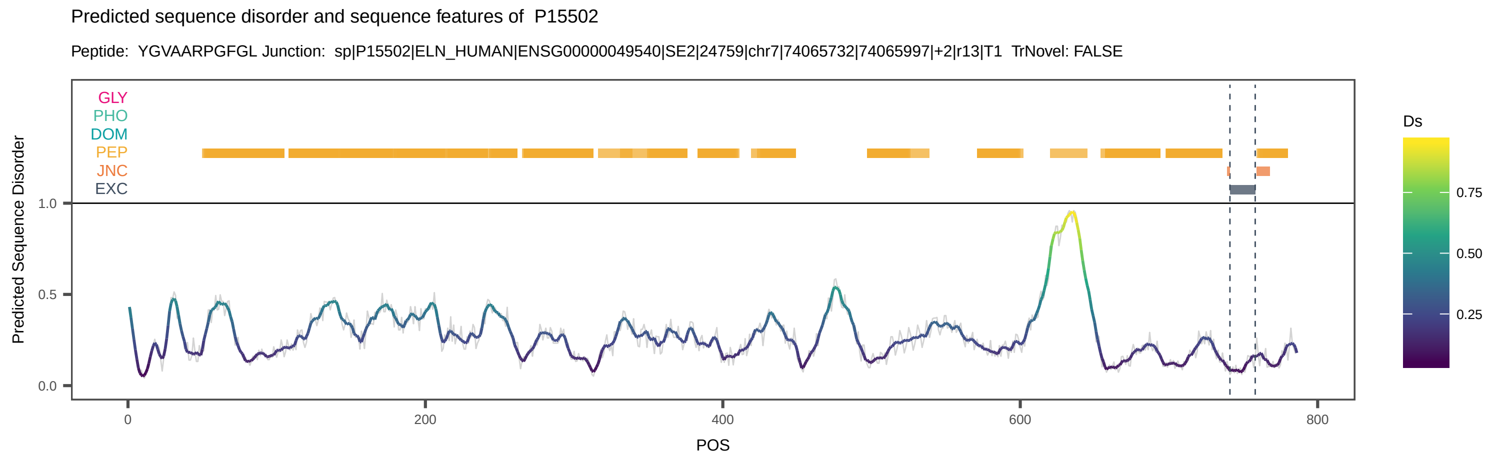

Predicted sequence disorder and sequence features of P15502
Peptide: YGVAARPGFGL Junction: sp|P15502|ELN_HUMAN|ENSG00000049540|SE2|24759|chr7|74065732|74065997|+2|r13|T1 TrNovel: FALSE
GLY
PHO
DOM
PEP
JNC
EXC
Predicted Sequence Disorder
1.0
0.5
0.0
0
200
400
600
800
POS
Ds
0.75
0.50
0.25

B

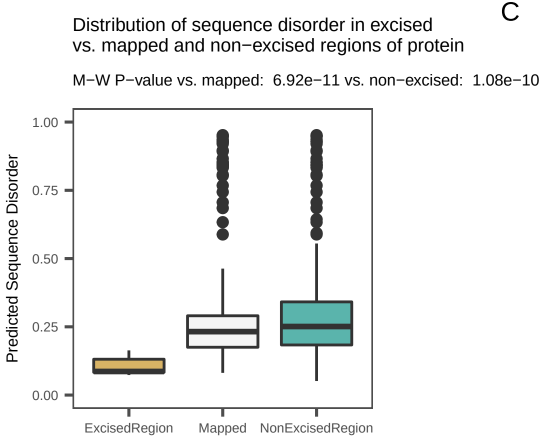

Distribution of sequence disorder in excised
vs. mapped and non−excised regions of protein
M−W P−value vs. mapped: 6.92e−11 vs. non−excised: 1.08e−10
Predicted Sequence Disorder
1.00
0.75
0.50
0.25
0.00
ExcisedRegion
Mapped
NonExcisedRegion

C
